# Nurses’ Attitudes Toward Lifelong Learning via New Technologies

**DOI:** 10.31372/20200502.1088

**Published:** 2020

**Authors:** Maria Lera, Kiriaki Taxtsoglou, Christos Konstanitinos Iliadis, Aikaterini Apostolos Frantzana, Lambrini Kourkouta

**Affiliations:** a European University of Cyprus, Nicosia, Cyprus; b G. Papanikolaou General Hospital of Thessaloniki, Thessaloniki, Greece; c International University of Greece, Thessaloniki, Greece

**Keywords:** nurses’ attitudes, lifelong learning, new technologies

## Abstract

**Introduction:** Lifelong professional education is considered as a qualitative indicator in the health discipline, as it can improve health professionals’ knowledge and skills, as well as nursing care.

**Purpose:** The purpose of this original research is to examine and record the attitudes and behavior of nurses working in state-run hospitals in the Municipality of Thessaloniki regarding lifelong education through new technologies. Identification of nurses’ motivations for lifelong distance education, recording of nurses’ perception of the need for continuing nursing education, and determining how nurses pursue lifelong learning are the objectives of this study.

**Methodology:** The study was conducted between January and March 2019. The sample of the study consisted of 124 nurses (*n* = 124) from three state hospitals of the Municipality of Thessaloniki. A questionnaire consisting of 5 parts was used as a research tool. SPSS 23 statistical software platform was used for statistical analysis.

**Results:** The sample consisted of 124 participants, 12 were men and 112 were women. The mean age of the participants was 42.37 years and the mean experience in the field was 16.78 years. Two main reasons for continuing education were attributed to the upgrade of the nursing profession and the need to improve the quality of care provided.

**Conclusions:** Nurses believe that continuing education is essential and their professional knowledge must periodically be enriched and renewed.

## Introduction

Lifelong professional education is considered as a qualitative indicator in the health discipline, as it can improve health professionals’ knowledge and skills, as well as nursing care. The municipality of Thessaloniki, which is the second largest city in Greece, lacks in continuing education while it must be harmonized with European standards (Koff, 2017). Thus, lifelong nursing education is considered vital, due to frequent changes in patient care, advances in knowledge, and technology, procedures, methods, guidelines, etc. (Dickerson, 2010).

Continuing education is one of the top European Union priorities in the field of education. Information and Communication Technologies (ICTs) pave the way for new, innovative lifelong education methods. Computer-based distance learning is a fast-growing type of education, and generally, a new way of providing education (Communities, 1998).

Continuing education is an all-encompassing term within a broad list of postsecondary learning activities and programs (Kipturgo, Kivuti-Bitok, Karani, & Muiva, 2014). Distance education is constantly being applied in the health sector as the internet and the world wide web have expanded many opportunities for flexible, convenient, and interactive education for health professionals (Cook, Levinson, & Garside, 2010). There is a wide range of traditional continuing education classes for nurses. There are classes requiring 24 contact hours every 1 or 2 years for nurses or 30 contact hours every 2 years and 400 practice hours every 4 years (Tachtsoglou, Lera, Iliadis, Frantzana, & Kourkouta, 2019).

Nurses may want to enroll in continuing education activities for a variety of reasons. Some may wish to improve their skills and abilities and the services they provide to their patients while others may simply want to gain more knowledge (Lera, Taxtsoglou, Iliadis, Frantzana, & Kourkouta, 2019). Furthermore, some nurses may seek personal benefits such as salary increases or promotions while others may want to feel more secure in their current professional positions (Tachtsoglou et al., 2019).

In some regions, particularly in rural areas professionals may face obstacles such as work schedules and commitments, lack of support (from colleagues, supervisors, and organizations), geographical distance, time-off from work, and additional costs while participating in continuing nursing educational programs (Shahhosseini & Hamzehgardeshi, 2015).

E-learning has many advantages since participants are not required to relocate. In addition, e-learning is often flexible and accessible, can be cost effective, and it allows trainees to learn at their own pace and from the location of their choice (Lera, Taxtsoglou, Iliadis, Frantzana, & Kourkouta, 2020). In addition, e-learning can provide customized content and teaching methods based on learners’ individual needs using a variety of multimedia such as text, sound, and even movement can be used to support knowledge acquisition and skills (Cook et al., 2010).

Attitudes and needs of culturally diverse populations such as Balkan countries, Asia, Pacific Islands, and the United States should be understood if research is to meet each community’s needs. Today this is made far easier than in the past, due to the internet. If surveys on e-learning are administered to culturally and linguistically diverse populations, academic researchers must first establish a relationship of trust and rapport with community partners that supports a safe space for communication (Nguyen-Truong et al., 2018). As a result, special designations of e-learning courses could be made available according to these health professionals’ needs (Vaona et al., 2018).

Computer-based distance learning is described as a dynamic, innovative, and successful way of providing learning opportunities. Learners can access a classroom through a website and participate in real-time lectures and group discussions (Kourkouta, Iliadis, Frantzana, & Vakalopoulou, 2018).

Training can also be provided asynchronously, where the learner can attend lectures and complete his/her work on his/her own time through the online platform or website (Simpson, 2003).

It is necessary for nurses be able to continue their education using appropriate methods in order to provide the patient with adequate and safe health care. Contrary to traditional learning methods, e-learning is a new flexible learning method for nurses. E-learning is expected to play a central role in providing continuing education to nurses (Cheng, 2013).

The purpose of this original research work was to identify and record the attitudes and behavior of nurses, working in state-run hospitals in the Municipality of Thessaloniki, toward lifelong education through new technologies.

The specific objectives were:

- Identifying and recording nurses’ motivations for lifelong distance education
- Identifying and recording nurses’ perception of the need for continuing nursing education
- Identifying the methods nurses use to pursue lifelong learning.

### Innovation Process—Upgrading Existing Knowledge

Very few studies have been conducted globally and especially, in countries with populations who reside mainly on islands such as Asia/Pacific Islands and Greece (Masri, 1999; Petaloti, 2009). These countries have a vital need for lifelong learning services via new technologies, because the majority of nurses in those countries stay and work long distances from each other (Ruggeri, Farrington, & Brayne, 2013). As a result, e-learning is the appropriate way for nurses to communicate with each other and share their views with both academics and colleagues. This service would enable nurses from around the country to share their valuable attitudes toward nursing and emergency situations that may arise on a daily basis (Ruggeri et al., 2013).

## Material and Methods

This cross-sectional study was conducted between January and March 2019. Initially, 127 questionnaires were distributed. However, three of them were inadequately filled and therefore were not taken into consideration during the analysis stage. The sample of the study consisted of 124 nurses (*n* = 124) from three state hospitals in the municipality of Thessaloniki while the initially calculated model sample was 120 participants. Fifty-one questionnaires were collected from Ippokrateio General Hospital of Thessaloniki, — 43 questionnaires were collected from University General Hospital of Thessaloniki AHEPA and 30 from Agios Dimitrios General Hospital. Ethical approval was obtained from the Scientific Councils of the hospitals. The participant-nurses took part in the study voluntarily and were previously informed about the purpose, the methods, and the procedure. The participants were informed that all provided data will remain confidential and will be used solely for the purpose of this study. Consent of the interested parties was obtained by one of the researchers after the verbal information about the type of survey and the questionnaire. The questionnaires were distributed by the same researcher during the morning shift because more nurses work in the morning; so, it was easier to collect the questionnaires. It took about 20 minutes for each nurse to complete the questionnaire and the researcher was present until the last questionnaire was completed by the participants.

Sampling bias is present in the study because the sample was collected in such a way that some members of the intended population had a lower or higher sampling probability than others and it was not a random sample.

A questionnaire consisting of five (5) parts was used as a research tool:

The first part consists of 24 questions aiming at exploring nurses’ perceptions of the need for continuing nursing education (questions 1–5), and their willingness to participate (questions 12–24), and their pursuit of continuing education (questions 6–11).

Questions 12–21 have been used in Shahhosseini and Hamzehgardeshi (2015) research to identify the expedition and difficulties that affect nurses’ intention to participate in continuing education programs. Reliability analysis was estimated with Cronbach’s alpha coefficient which was 0.92.

Questions 1–11 and 22–24 have been used in Mouriki’s (2015) research to delve into the desire and need for continuing education of nursing staff. The Cronbach’s alpha factor depicts reliability or internal relevance so as to check if the questions have been asked correctly and answered properly. The alpha indicators in the present research are very good. It means that the questions-answers have a very good relevance.

The second part (questions 25–54) consists of the Participation Reasons Scale (PRS), which is a 7-point Likert-type scale that outlines the reasons (incentives) for professionals to pursue ongoing educational programs. The scale has been used many times to investigate the motivations of various professional groups for continuing education programs. The questionnaire has been translated, adjusted, and weighted in Greek by Panagiotopoulou et al. (2016). It has also been applied to health professionals (military doctors and nurses) by the same researchers and has been tested with the Cronbach’s alpha coefficient. For nurses, the Cronbach’s alpha coefficient was 0.93. This means that this tool, as developed, provides high reliability for the collection of data related to nurses’ motivation to pursue continuing nursing education programs.

The third part of the questionnaire consists of 8 questions (questions 55–62) which aim to record the level of usage and familiarity of the respondents toward computers and seven questions (questions 63–69) aiming to delve into the attitude of the respondents toward the use of computers in their workplace. Similar questions regarding the level of usage and familiarity of respondents toward computers have been used in the studies of Kipturgo et al. (2014) and Brumini et al. (2005). The questions aiming at delving into respondents’ attitude toward computer use in their workplace have been used in the study by Stricklin, Bierer, and Struk (2003) and Brumini et al. (2005).

Reliability analysis was performed based on Cronbach’s alpha coefficient of 0.90. In the questionnaire, only 7 of the 20 sentences used by the NATC tool were used. Three were positively worded and 4 were negatively worded out of the 7 sentences. The 5-point Likert scale was used, as well as the NATC tool (Brumini et al., 2005; Kipturgo et al., 2014; Stricklin et al., 2003).

Positive worded sentences include Questions 63, 65, and 69, with answers ranging from 5 to 1 represented by the answers “I totally agree”, “I agree”, “I do not agree/disagree”, “I disagree”, and “I disagree absolutely”. The higher the agreement rate was, the greater the degree of agreement was. Question-statements 64, 66, 67, and 68 include negative worded sentences that are scored in the opposite order in comparison with those of positive worded sentences.

The fourth part consists of the attitude scale concerning web-based learning used by Yu and Yang (2006) in their research. It is like a 5-point Likert scale. This scale consists of 16 question-statements, seven (7) negative and nine (9) positive ones. Negative statements include question-statements 77 and 80–85. The rest of the question statements regard positive statements. Positive statements have a response range of 5–1 represented by the responses “strongly agree”, “agree”, “neutral”, “disagree”, and “strongly disagree”. The higher the degree is, the greater the degree of agreement. Negative statements were scored in the opposite way. The range of overall scores could range from 16 to 80 points, with higher scores indicating that nurses had a more positive attitude toward computer-based distance education.

Lastly, the fifth part contains 6 demographic questions and includes questions regarding gender, age, marital status, position, work experience, and educational level.

The questionnaire used in this study consists of 5-point and 7-point Likert-type scales. Likert-type scales are often used as they do not only record the general agreement or disagreement with a sentence but also the degree of agreement (Zafiropoulos, 2005).

SPSS version 23 statistical package was used for analysis. Nonparametric tests of the equality of continuous, one-dimensional probability distributions were used to compare a sample with a reference probability distribution (Kolmogorov–Smirnov *Z* test) and analyze group differences when the dependent variable is measured at a nominal level (Chi square test). Kruskal–Wallis one-way analysis of variance was used for comparing two or more independent samples of equal or different sample sizes. It analyzed the demographic information of the sample and then the answers of the participants derived from the questionnaire.

The statistical analysis was adjusted for each research parameter which was as follows:

- Degree of satisfaction by age group.
- Degree of satisfaction by gender.
- Degree of satisfaction by years of work experience.
- Degree of satisfaction by the marital status of the participant.
- Degree of satisfaction by the level of the participant’s education.

Descriptive statistics were used to analyze the demographics.

## Reliability Analysis

The internal consistency of the questionnaire was assessed using the Cronbach’s alpha coefficient. The index for the overall questionnaire (Likert scales) is 0.97. For the individual scales the alpha coefficient is:

For the factors that influence participation in continuing nursing education programs, *a* = 0.93.

The importance of the reasons for participating in continuing education programs, *a* = 0.99.

The attitude toward computer use, *a* = 0.91.

There is a high reliability index of the research tool derived from the calculations above, which indicates that reliable conclusions can be drawn from the research. Cronbach’s alpha is grounded in the “tau equivalent model” which assumes that each test item measures the same latent trait on the same scale (Tavakol & Dennick, 2011). Therefore, if multiple factors/traits underlie the items on a scale, as revealed by factor analysis, this assumption is violated and alpha underestimates the reliability of the test.

## Results

The sample included 124 participants. The participation response rate was 97.63%. Of the participants, 12 were males (9.7% of the total) and 112 were females (90.3%). The mean age of participants was 42.37 years (TA = 8.14) and the mean years of work experience was 16.78 years (TA = 8.41). Regarding marital status, the majority of the sample were married (67 participants, 54% of the sample), 33 were single (26.6%), 13 participants (10.5%) were divorced, and 8 (6.5%) were widows/widowers, while 3 (2.4%) did not respond at all. Also, most participants (113 persons, 91.1%) were staff nurses and 8 (6.5%) were head nurses. The educational level of approximately 50% of participants (68 persons, 54.8%) were university graduates, 36 persons (29%) had two-year graduate degree, and 20 participants (16.1%) had a master’s degree.

**Figure 1.**
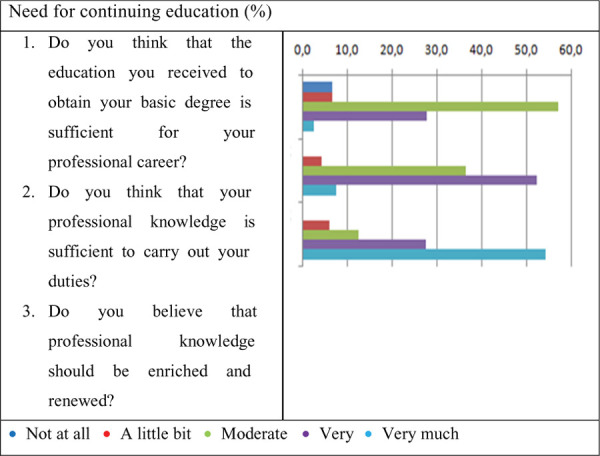
Need for continuing education.

Participants’ responses regarding the need for continuing education show a moderate to high degree of agreement that the training they received to obtain a basic degree is sufficient for vocational training. Specifically, 70 participants (56.5%) answered they moderately agreed and 34 participants (27.4%) strongly agreed. The degree of agreement that professional knowledge is sufficient to perform the tasks of the participants was quite high: 63 people (50.8%) answered that it was sufficient and 44 people (35.5%) stated it was moderate preparation. However, the largest proportion of the sample responded “very much” to the fact that professional knowledge needed to be enriched and updated (65 people, 52.4%) (Figure 1).

Ninety-one participants (73.4%) answered positively to the question: “Do you think continuing nursing education should be compulsory for employees?” while the remaining 33 participants (26.6%) answered negatively.

Regarding the reasons for compulsory continuing education, 90 participants (78.9% of the sample) responded that an important reason for continuing education is the upgrade of the nursing profession; it was the most popular response. The next most frequent response was the “need to improve quality of care” with 83 responses (72.8% of the sample), followed by “technology development” (76 responses, 66.7% of the sample), and finally other reasons such as competition, insufficient degree of knowledge, and additional security (68 answers). Sixty-four participants (51.6%) provided all the answers.

Eighty-three participants (66.9% answered yes, while 41 [33.1%] answered that they did not participate) provided an answer to the question “Have you participated in continuing vocational training programs in the past?”.

When asked when was the last time they participated in a continuing education program, 27 people (21.8%) answered “this year”, 21 people (16.9%) answered 1 year ago, 21 people (16.9%) responded 2 years ago, 8 people (6.5%) replied 3 years ago, and 27 people (21.8%) answered more than 3 years ago. The answers show that about one quarter of the participants have attended training programs recently (Table 1).

**Table 1:** Time Period the Participants Took Part in a Continuing Education Program

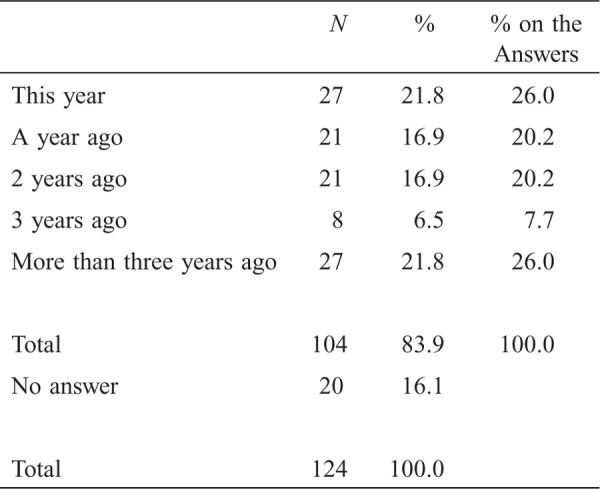

Regarding the question about the origin of the training initiative, 42 participants (33.9%) responded on their own initiative, 21 participants (16.9%) responded on the initiative of the institution, and about half answered (53 participants, 42.7%) were at the initiative of both (Table 2).

**Table 2:** Comparison of Participants’ Own Initiative to be Further Educated vs. Their Employer’s Initiative

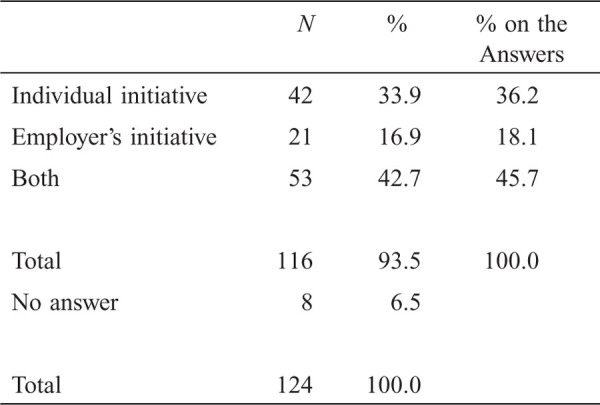

Regarding the question “Do you feel the need to be educated and further your education either in the field you are currently working in or in another field?”, the majority of respondents (97 people, 78.2%) answered positively. For the question: “Do you have the desire to train and further your education either in the field you are currently working in or in another field?”, 99 participants (79.8%) answered positively. Regarding the timing of continuing education programs, 90 people (72.6%) stated that they prefer completing continuing education courses during working hours and 32 people (25.8%) outside of working hours, while 2 people did not answer this question.

**Figure 2.**
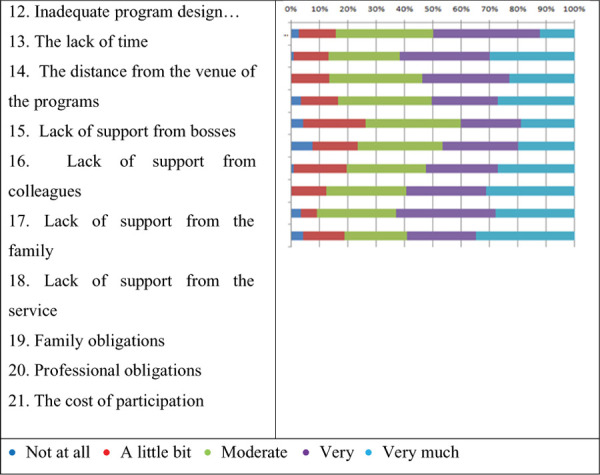
How much would the factors above affect your participation in continuing education programs?

Regarding the questions that delved into the extent to which respondents would be influenced by the participation of continuing education programs in a series of factors, the answers are given in Figure 2 and Table 3. The answers indicate the most influential factors were the cost of participation (59.4% answered much or too much), lack of time (61.8% answered much or too much), occupational obligations (63.1% answered much or too much), and family obligations (59.5% responded much or too much). In addition, more than 50% (much or very much) replied “Distance from the place where courses take place” (53.7%), “Lack of support from the workplace” (52.2%), and “The lack of support from managers” (50.4%). Less impact was attributed to factors such as “Inadequate planning of education programs”, “Lack of peer support”, and “Lack of support from the family”.

**Table 3:** Frequency of the Organization of Educational Programs by Working Departments

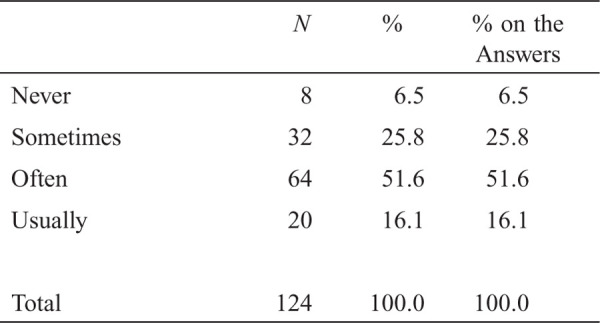

Regarding the question “How often are educational programs organized by your service?” the dominant answer was “often” (64 participants, 51.6%) (Table 3).

Regarding the question “How satisfied are you with the content of those programs?”, the dominant answer was “moderately” (70 participants, 56.5%) (Table 4).

**Table 4:** Degree of Satisfaction with the Content of those Educational Programs

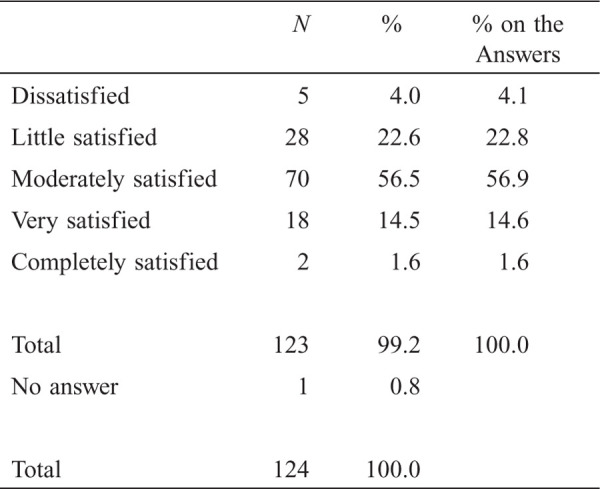

Regarding the question “Is your service considered to enable employees and provide them with opportunities for education and training?”, 50 participants (40.3%) answered moderately, 38 participants (30.6%) answered slightly, and 27 (21.8%) did not answer at all (Table 5).

**Table 5:** Degree of Enabling Employees and their Provision with Opportunities for Education and Training by the Service

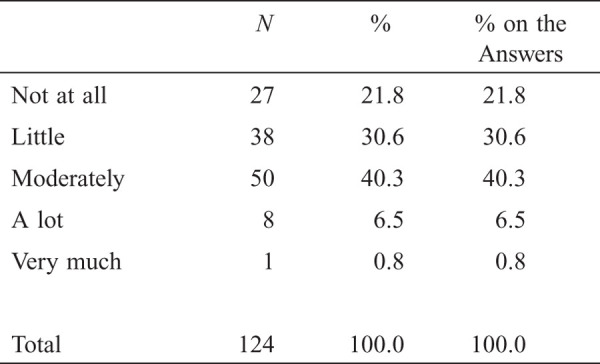

### Searching Incentives for Continuing Education

The questionnaire section examining how important are your reasons for participating in continuing education programs included 30 statements. Exploratory factor analysis (EFA) was performed to group the 30 statements, using Principal Component Analysis, which calculated two factors that accounted for 78.3% of the total scale variance, with a sampling rate = 0.947. The resulting factors have high loads and very good internal consistency:

Factor 1: Improving the profession and position as a nurse = 0.980, and

Factor 2: Reflection on the role and profession of the nurse and interactions with colleagues, *a* = 0.975.

For all statements/incentives percentages were high, between 5.32 and 5.79 on the 7-point Likert scale. Although the differences in the mean of the responses were small, the type of motivation for the highest mean was “Keeping up with new developments in nursing” (Mean = 5.79, SD = 1.314), followed by “Increasing the probability of progress in a career” (Mean = 5.76, SD = 1.324), after the motivation of “Becoming more skilled in my work” (Mean = 5.74, SD = 1.384), and then “Developing professional skills necessary to maintain high quality work” (Mean = 5.65, SD = 1.355).

The mean values of the responses to the factor referring to nurses’ reflection on their profession, the value of their duties, their commitment to it, their role, and relationship with their colleagues were at lower levels (between 5.15 and 5.47) than the first factor, but also high. The highest mean was “Improving my individual offering to the public as a health nurse” (Mean = 5.47, SD = 1.351), followed by “Maintaining my identity through my profession” (Mean = 5.39, SD = 1.429), and “Responding to the cognitive challenges of my fellow healthcare workers and nurses” (Mean = 5.37, SD = 1.451). Next was “Reflect on the value of my nursing duties” (Mean = 5.34, SD = 1.319). Finally, the motives of “Exchanging views with colleagues” (Mean = 5.23, SD = 1.291), “Considering the limitations of my role as a nurse” (Mean = 5.18, SD = 1.426), and “Being able to relate my professional views to those of my colleagues’ views” (Mean = 5.15, SD = 1.343).

### Familiarity with Computers and Usage

The third part of the questionnaire was about familiarity with computers and applications and their usage. When asked if they had taken computer skills courses in their basic education, more than half of the participants (71 people, 57.3% of the total sample) answered that they had no relevant education in basic computer education. When asked if they use a computer (and where), 39 participants (31.7%) answered that they use one at home, 13 participants answered that they use one at work (10.6%), 57 participants responded at home and at work (46.3%), and 14 participants (11.4%) responded they did not use one at all.

Regarding the number of hours participants use a computer, the dominant group of participants used the computer 1–3 hours per day (49 people, 41.9%) (Table 6).

**Table 6:** Distribution of Participants by Computer Usage Time

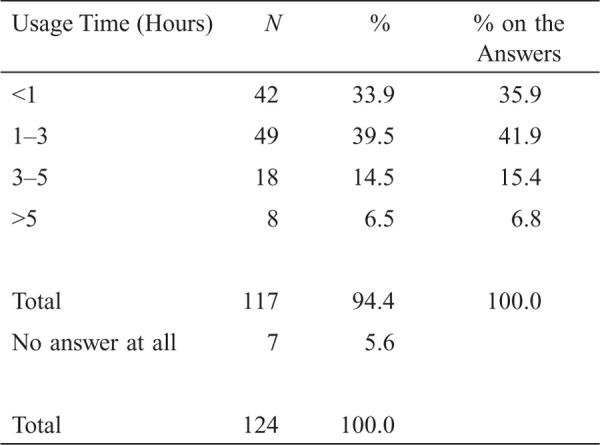

On the question “The main reason you use the computer” (multiple answers), 6.6% of the sample answered they do not use a computer at all, 56.2% use a computer for working purpose, 53.7% for communication, 39.7% for entertainment, and 55.4% for education (Table 7).

**Table 7:** Reasons for Using a Computer

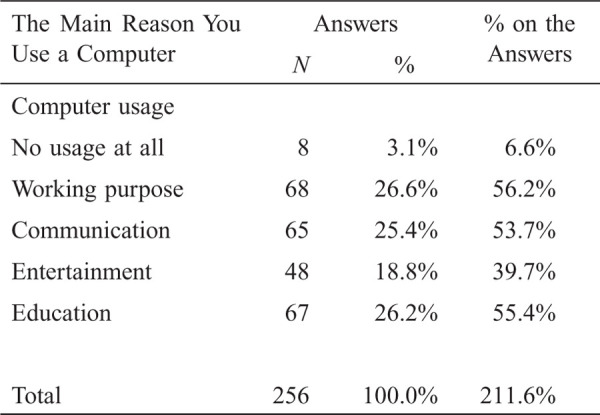

Sixty-six percent of the sample responded positively to the question: “Do you think you can handle the word processor comfortably?”. Sixty percent responded that they were familiar with the question: “Are you familiar with computer terminology?”. Fifty-seven percent answered positively to the question “Do you think you can prepare and use Power Point presentations?”, and 89% responded positively to the question “Do you have an email address?”.

**Figure 3.**
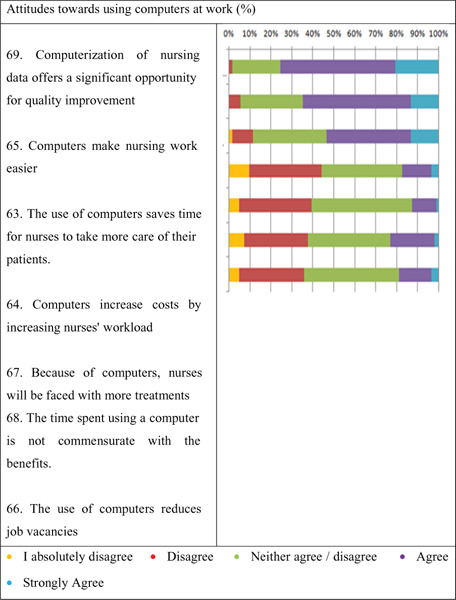
Attitudes toward using computers at work.

The next set of questions (7 statements) explored participants’ attitudes toward the use of computers in the nursing profession. The general attitude of the sample respondents were positive as the means of the answers were between 3.19 and 3.94 on the five-point scale. The mean for all statements was 3.46. Specifically, the dominant answer was that the participants agreed (54.5%) to the statement “Computerization of nursing data offered a significant opportunity to improve the quality of care provided”. The dominant answer was the participants agreed (51.2%) to the statement “Computers make nurses work easier”; and for the statement: “Computer use saves time for nurses to take more care of patients”, the percentage of those who agreed was 40.2% and those who were neutral was 35.2% (Figure 3).

Regarding the negative attitude on the statement “Computers increase costs by increasing the workload of nurses” the main answer was “Neither agree/nor disagree” (38.5%) followed by disagreement (34.4%). For the statement: “Nurses will face more treatments due to computers”, the prevailing answer was “Neither agree/nor disagree” (47.9%), followed by the answer “I disagree” (34.7%). Regarding the statement: “Time spent using the computer is not commensurate with the benefits”, the dominant answer was “Neither agree/nor disagree” (39.3%) followed by disagreement (30.3%). Finally, the statement: “Computer use reduces job vacancies”, the main answer was “Neither agree/nor disagree” (45.1%), followed by the answer “I disagree” (31.1%).

### Delving into the Attitude toward Distance Continuing Education

Nurses’ attitudes toward distance education were assessed by analyzing the 16 statements in the last part of the questionnaire that used a 5-point Likert scale. The results revealed participants demonstrated a positive attitude toward distance learning through new technologies. The mean values of responses to positive statements ranged from 3.25 to 4.08, on a five-point scale, and negative statements from 3.21 to 3.30. The overall mean for all responses was 3.56. These figures indicate an above average response. The statements that received the highest level of agreement from the participants were: “It allows me to learn freely according to my free time” (predominant answer is “I agree” with 62.1% of the sample, Mean = 4.08, SD = 0.670); the statement “Saves time moving from/to continuing education programs place” (predominant answer “I agree” with 63.7% of the sample, Mean = 4.02, SD = 0.643); and the statement “I am allowed to choose the courses I want to attend” (predominant answer “I agree” with 63.4% of the sample, Mean = 3.97, SD = 0.680).

## Correlations

In this section, correlations between participants’ responses and their characteristics are made. The significance level for statistical tests is 95% (*P* < 0.05), and the tests to be performed are nonparametric, as the variables representing respondents’ responses are not normally distributed (Kolmogorov–Smirnov *Z* test for normal distribution, *P*-value < 0.05).

Attitudes on the necessity of continuing education did not differ among the three state hospitals of the Municipality of Thessaloniki (Kruskal–Wallis test, *P* > 0.05).

Concerning the organization and satisfaction of training programs (questions 22, 23, and 24), these data did not vary according to the hospital in which the participants worked.

The Kruskal–Wallis test was used on the two motivation factors created to find out whether nurses’ motivation to pursue continuing nursing education programs differs depending on participants place of employment. The test showed that there was no statistically significant difference in motivation among employees of different hospitals (*X*^2^ = 1.267, df = 2, *P* = 0.613).

Similarly, nurses’ attitudes toward computer usage in their work did not differ in comparison with the hospital in which they worked (Kruskal–Wallis test, *P* > 0.05).

Concerning participants’ attitudes toward distance education through new technologies, the responses to just one statement “It makes me feel isolated from the teacher and classmates” on the scale (out of 16 statements) varied significantly, depending on the hospital in which they work (*X*^2^ (2) = 7,450, *P* = 0.024).

### Comparison by Gender

Male nurses’ opinions did not differ significantly from those of women on the need for continuing education (Mann–Whitney (*U*) = 566.000, *Z* = −957, *P*-value = 0.339).

Regarding the factors that influence nurses’ participation in continuing education programs, two out of ten factors differed according to the gender of the respondents. Family responsibilities was one of the two factors (*U* = 318.00, *Z* = −3.033, *P* = 0.002), for which women are statistically more affected (Mean Rank = 64.08) than men (Mean Rank = 33.00). The second factor was occupational obligations (*U* = 402.00, *Z* = −2.322, *P* = 0.020), for which women were also more affected (Mean Rank = 63.85) than men (Mean Rank = 40.00).

The difference between men and women was significant (*U* = 424.00, *Z* = −2.2286, *P* = 0.022) regarding the organization of educational programs and satisfaction derived from them, with women giving more positive answers (Mean Rank = 64.71) than men (Mean Rank = 41.83). The views for the content of the programs (question 22) and the views on whether the service offers facilities and opportunities for education and training (question 23), did not significantly differ statistically between men and women regarding respondents’ motivation to participate in continuing education programs.

Women responded statistically significantly more than men (*U* = 389.50, *Z* = −2.358, *P* = 0.018), that improving their profession is one motive for participation. (Mean Rank for women = 64.49 and Mean Rank for men = 38.96).

The attitude toward computer use at work did not differ between men and women (*P* > 0.05 for all scale statements). Moreover, the attitude toward distance education through new technologies did not differ between men and women (*P* > 0.05 for all scale statements).

### Correlation to Age

Age was negatively correlated with responses to some statements regarding the use of computers, and distance education through new technologies. Specifically, a nonparametric Spearman’s Rho correlation test was found to indicate that age of respondents was negatively correlated with attitudes toward technology use expressed by the questions “Computers make nurses’ work easier” (*r*s = −0.28, *P* = 0.001), “Nurses will face more treatments because of computers” (*r*s = −0.190, *P* = 0.038), and “Computerization of nursing data offers a significant opportunity to improve the quality of care provided” (*r*s = −0.191, *P* = 0.038). Also, respondents’ age was negatively correlated with attitudes toward distance education through new technologies expressed by the questions “It can provide me with more learning information” (*r*s = −0.233, *P* = 0.010) and “It will increase costs software and hardware on my computer” (*r*s = −0.238, *P* = 0.008). The negative correlation coefficient for these questions was indicative that older nurses tend to have a less positive attitude toward computer use and distance education.

### Comparison According to Education Level

There was no statistically significant difference concerning the views on the necessity of continuing education among the three levels of education: two-year graduates, university graduates, and postgraduates (Kruskal–Wallis test, *P* > 0.05).

Also, there was no statistically significant difference concerning the extent to which various factors influence participation in continuing education among the three levels of education (Kruskal–Wallis test, *P* > 0.05).

Regarding the organization and satisfaction of educational programs, the level of education did not significantly differentiate the answers (Kruskal–Wallis test, *P* > 0.05).

Concerning the incentives to participate in nursing education programs, the Kruskal–Wallis test showed significant differences on how motivated participants are to improve their profession and position, depending on their level of education (*X*^2^ (2) = 6,026, *P* = 0.049). The highest scoring team was postgraduates (Mean Rank = 79.76), followed by two-year graduates (Mean Rank = 61.94) and finally university graduates (Mean Rank = 57.07).

Concerning nurses’ attitudes of the sample toward the use of computers at work in comparison with the level of education, it was found in three out of seven statements that the educational level was significantly differentiated. Specifically, for the statement “Nurses will be confronted with more treatments due to computers” there was a significant difference (*X*^2^ (2) = 6,727, *P* = 0.035). Postgraduates recorded the most positive attitude (Mean Rank = 75.60), followed by university graduates (Mean Rank = 61.27) and lastly, by two-year graduates (Mean Rank = 52.16). Regarding the statement “Time spent using a computer is not commensurate with the benefits”, there were significant differences (*X*^2^ (2) = 7,455, *P* = 0.024), postgraduates had the most positive attitude (Mean Rank = 73.50), followed by university graduates (Mean Rank = 64.30), and finally two-year graduates (Mean Rank = 49.71). Regarding the statement “Computerization of nursing data offers a significant opportunity to improve the quality of health care provided” the differences between groups were significant (*X*^2^ (2) = 18,838, P < 0.001), with postgraduates (Mean Rank = 88 45) being significantly different from university graduates (Mean Rank = 57.75) and two-year graduates (Mean Rank = 51.63).

The level of education was also tested to determine whether it influenced respondents’ attitudes toward distance education through new technologies. Those participants with higher levels of education had a more positive attitude toward computer use.

### Brief Summary

There was a statistically significant difference (*X*^2^ (2) = 19,944, *P* < 0.001) for the statement “It can offer new nursing knowledge”, with postgraduate students having a more positive attitude (Mean Rank = 90.45), followed by two-year graduates (Mean Rank = 61.57), and finally university graduates (Mean Rank = 54.77). There was a statistically significant difference (*X*^2^ (2) = 15,941, *P* < 0.001) for the statement “It saves time moving from/to continuing education programs”, with postgraduate students having a more positive attitude (Mean Rank = 83.58), followed by two-year graduates (Mean Rank = 67.43), and finally university graduates (Mean Rank = 53.69).

For the statement “It allows me to choose the courses I want to attend”, nurses had significantly (*X*^2^ (2) = 11,393, *P* = 0.003), more positive attitude (Mean Rank = 80.73), followed by two-year graduates (Mean Rank = 64.74), and finally university graduates (Mean Rank = 54.94).

Postgraduate nurses had a significantly (*X*^2^ (2) = 7,626, *P* = 0.022) more positive attitude (Mean Rank = 80.15), followed by two-year graduates (Mean Rank = 62.61), and finally university graduates (Mean Rank = 57.25) for the statement “It is a cost-effective education method”.

For the statement “It is not useful to improve individual care ability”, postgraduates had a significantly (*X*^2^ (2) = 17,579, *P* < 0.001), more positive attitude (Mean Rank = 75.18), followed by university graduates (Mean Rank = 67.82), and finally two-year graduates (Mean Rank = 42.51).

For the statement “Computer learning will reduce learning outcomes”, postgraduates appeared to disagree with a significant difference (*X*^2^ (2) = 8,134, *P* = 0.017) (Mean Rank = 79.50), followed by university graduates (Mean Rank = 61.43), and finally two-year graduates (Mean Rank = 53.35).

For the statement “It will increase my computer software and hardware costs” postgraduates appeared to disagree with a significant difference (*X*^2^ (2) = 6,690, *P* = 0.035) (Mean Rank = 79.63), followed by university graduates (Mean Rank = 59.46), and finally two-year graduates (Mean Rank = 56.94).

## Discussion

The results of the survey showed that most participants strongly agree on the necessity of continuing education. The reasons for compulsory continuing education are attributed to the upgrade of the nursing profession and to the need to improve the quality of care provided. Participants disagreed that compulsory continuing education is necessary because of the development of technology, but this is of a lesser priority than the previous two reasons.

The main factors that appeared to affect nurses’ participation in continuing education programs were lack of support from supervisors (95.8%), program funding (94.8%), and family obligations (94.8%) (Adamu, 2015).

A survey about lifelong training conducted for teachers in Cyprus who are in-service found that the main reason for their participation was the issue of improving their performance at work, with the main obstacles being the lack of time and inadequate and insufficient information (Kourkouta et al., 2018).

Concerning the participants’ experience in training programs, more than two out of three responded they have already participated, either recently or in the past.

The need and desire of participants to be additionally educated in their subject or elsewhere was intense as about eight out of ten responded positively, expressing, at the same time, a majority wishing to be educated during working hours.

The factors that had the greatest impact on respondents’ participation in continuing education programs mainly were lack of time, occupational and or family obligations, and distance from the programs taking place. Others included lack of support from both the workplace and managers; to a lesser extent, factors such as inadequate planning of education programs; lack of support from colleague; or lack of support from family affect nurses.

Concerning the organization of training programs, the results showed they are organized at a moderate frequency, the trainees are moderately satisfied, and the nursing service does not offer special facilities and opportunities for training.

In a survey conducted by the Hellenic Confederation of Professionals, Craftsmen & Merchants (GSEVEE) and the Labour Institute of the Greek General Confederation of Labour (INE-GSEE), it was found that 81.84% was the major percentage referring to participation costs. “Lack of information on programs” was the second factor that prevented participation and the “Lack of time due to work obligations” was the third factor. Almost a quarter of participants stated that facilitating their work would promote their participation (Karalis, 2013).

Participants’ responses showed that every motive was significant and scored higher than the average response regarding the questions: “Keeping up with new developments in Nursing”, “Increasing my career prospects”, “Becoming more skilled (or) at work”, “Developing professional skills necessary to maintain high quality work”, “Develop new professional knowledge and skills”, and “Feel more secure in this position”. For the second factor, the statements that included the strongest motivation were “Improve my individual offer to the public as a health nurse”, “Maintain my identity through my profession”, “Respond to my colleagues’ cognitive challenges”, “Reflect on the value of my nursing duties”, “Learn through interaction with other nurses”, and “Evaluate the direction my profession has taken”.

In the study of Inouye and Alpert (2018) it is stated that evaluation of healthcare workers’ strengths and challenges may be enhanced by working with an educator or taking a course either conventional or e-learning. More disaggregated data for specific ethnic groups such as nurses come from Balkan countries and Asian and Pacific countries would offer valuable facts for research, practice, and educational needs of the population (Inouye & Alpert, 2018).

A survey conducted by the Hellenic Confederation of Professionals, Craftsmen & Merchants (GSEVEE), and the Labour Institute of the Greek General Confederation of Labour (INE-GSEE) found that the most important incentives for participation were factors about “professional upgrading” and “interest in learning” groups. Specifically, 90% of the respondents “like to learn new things”. Also, factors such as “being more efficient in my job” had a high level of selection of 86.4% (Karalis, 2013).

In the study by Adamu (2015), the most important incentives for continuing education of nurses recorded were the development of skills needed to meet patient needs (95.8%), improved self-efficacy (95.8%), and information on recent developments in nursing science (86.5%) (Adamu, 2015).

In terms of computer use, only a small percentage did not use a computer, at home or at work, or in both. Most nurses used the computer one to three hours a day for work, communication, training, and less for fun. More than half the nurses could handle the Word processor comfortably and prepare and use Power Point presentations. They are familiar with computer terminology, and about nine out of ten have email addresses. Respondents’ attitudes toward using computers at work were above average.

Surveys in Serbia and Turkey also showed a positive attitude toward computer use by nurses and even asked nurses to support their service in order to expand their knowledge and skills regarding computer use (Gürdaş Topkaya & Kaya, 2015; Milutinović, Ćirić, & Simić, 2018).

Similarly, participants’ attitudes toward distance learning through new technologies were recorded as positive. The most positive points highlighted by the participants’ responses were flexibility and time saving (“It allows me to learn freely in my own time” and “saves time moving to/from continuing education programs”) as well as the choice (“It allows me to choose the courses I want to attend”).

Nurses’ attitude toward distance education continuing education was encouragingly positive in two studies (Chong et al., 2016; Karaman, 2011). Also, in the study of Uslu, Buldukoglu, and Zayim (2014) nurses appeared to be willing to participate in distance learning programs at a rate of 87.1%.

Participants’ views on the need for continuing education did not differentiate significantly between the two genders, as their attitude toward the use of computers at work and distance education does through new technologies.

There seem to be factors that influence participation in educational programs where women were more likely than men to think that family and occupational obligations was a factor that influenced them. Given the role of women in the family and their role as mothers, it is understood that they are entrusted with increased responsibilities that require them effort and time, and thus act as inhibitors in education (Uslu et al., 2014).

Similar results were found in the study by Ni et al. (2014) regarding respondents’ motivation to participate in continuing education programs, and women responded statistically significantly more than men that improving their profession is an incentive (Ni et al., 2014).

Age was statistically negatively correlated with responses to some statements regarding the use of computers, and distance education through new technologies (“Computers make nurses’ jobs easier”, “Nurses will face more treatments due to computers”, “Nursing computerization offers significant opportunity to improve quality of care provided”, “Training can provide more learning information”, “It will increase computer software and hardware costs”). Older nurses tend to have a less positive attitude toward PC use and distance education (Kourkouta et al., 2018).

The age of nurses was a factor in differentiating nurses’ attitude toward computer use and was supported by Kipturgo et al. (2014), with nurses under 40 having a more positive attitude than those older than 40 (Kipturgo et al., 2014).

What is more, there was a significant statistical difference in nurses’ attitudes regarding age, marital status, education, job, computer experience, computer lifetime, and computer use in Kaya’s (2011) research.

The level of participants’ education (two-year graduates, university graduates, and postgraduates) did not differ in the views on the necessity of continuing education, the views on the organization, and satisfaction of educational programs or the factors that influence participation in continuing education programs. On the other hand, incentives were differentiated with postgraduates expressing greater motivation to improve their profession and position than two-year graduates, and finally university graduates. Postgraduates also expressed more positive attitudes toward computer use and distance learning through new technologies.

The level of education appeared to influence nurses’ attitude toward computer use in Kipturgo et al. research (2014) where those with a higher level of education had a more positive attitude toward computer use (Kipturgo et al., 2014).

## Conclusions

Although nurses believe the education they received for their basic degree was sufficient for vocational training, and that their knowledge was sufficient to carry out their duties, they thought it was necessary that professional knowledge should be enriched and renewed. Moreover, about three out of four nurses agreed that continuing nursing education should be compulsory for health workers.

Regarding nurses’ motivation to participate in training programs, exploratory factor analysis identified two factors. The first factor related to incentives to improve the nurse’s profession and position, such as better responding to knowledge or skills in the practice, developing new professional knowledge and skills, improving productivity and quality, professional advancement, expanding the visual and coping with developments in the field, job security, better and more effective patient service, and personal benefits. The second factor called reflection on the role and profession of the nurse and interaction with colleagues included elements such as nurses reflecting on the value of their nursing duties, examining the limitations of the nursing role, evaluating the course of their profession, reinforcing the image of the profession, redefining their commitment to the profession and their duties, interacting and exchanging views with colleagues, and preserving their identity through their work.

There was also a positive attitude by nurses regarding their attitudes toward distance education through new technologies. Creating a learning environment that supports, motivates, and improves nurses’ participation in continuing nursing education is likely to lead to more positive cognitive, emotional, and behavioral outcomes. In addition, there is evidence that self-motivated nursing staff engaged in educational activities will provide more and better patient-centered care, as well as being more supportive and more cooperative.

## Acknowledgements

Lera Maria: RN, MSc, General Hospital “Ippokratio” Thessaloniki, Greece. Taxtsoglou Kiriaki: RN, MSc General Hospital “G. Gennimatas” Thessaloniki, Greece. Iliadis Christos: RN, Private Diagnostic Health Center of Thessaloniki, Greece. Frantzana Aikaterini: RN, MSc, PhD Candidate, European University of Cyprus, Nicosia, Cyprus & General Hospital “Papanikolaou” of Thessaloniki, Thessaloniki Greece. Kourkouta Lambrini: Professor, Nursing Department, International University of Greece, Thessaloniki, Greece

## Declaration of Conflicting Interests

The authors declared no potential conflicts of interest concerning the research, authorship, or publication of this article.

## Funding

The authors received no financial support for the research, authorship, and/or publication of this article.
